# Novel MAGL Inhibitors Alleviate LPS-Induced Acute Kidney Injury by Inhibiting NLRP3 Inflammatory Vesicles, Modulating Intestinal Flora, Repairing the Intestinal Barrier, and Interfering with Serum Metabolism

**DOI:** 10.3390/molecules28217245

**Published:** 2023-10-24

**Authors:** Haixin Xiang, Yangui Wang, Lan Yang, Mingfei Liu, Chenghong Sun, Yuchao Gu, Jingchun Yao

**Affiliations:** 1 School of Medicine and Pharmacy, Ocean University of China, Qingdao 266003, China; xianghaixin1225@163.com (H.X.);; 2 School of Pharmacy, Shandong University of Traditional Chinese Medicine, Jinan 250355, China; 3 School of Chinese Materia Medica, Tianjin University of Traditional Chinese Medicine, Tianjin 301617, China; 4 State Key Laboratory of Integration and Innovation of Classic Formula and Modern Chinese Medicine, Linyi 276005, China; sch658@163.com

**Keywords:** AKI, inflammatory, NLRP3, metabolism, intestinal barrier, gut microbiota

## Abstract

Acute kidney injury (AKI) is a complication of a wide range of serious illnesses for which there is still no better therapeutic agent. We demonstrated that M-18C has a favorable inhibitory effect on monoacylglycerol lipase (MAGL), and several studies have demonstrated that nerve inflammation could be effectively alleviated by inhibiting MAGL, suggesting that M-18C has good anti-inflammatory activity. In this study, we investigated the effect of M-18C on LPS-induced acute kidney injury (AKI), both in vivo and in vitro, by using liquid chromatography-mass spectrometry (LC-MS), 16S rRNA gene sequencing, Western blot, and immunohistochemistry. The results showed that both in vivo and in vitro M-18C reduced the release of TNF-α and IL-1β by inhibiting the expression of NOD-like receptor thermal protein domain-associated protein 3 (NLRP3) and apoptosis-associated speck-like protein containing a CARD (ASC) protein; in addition, M-18C was able to intervene in LPS-induced AKI by ameliorating renal pathological injury, repairing the intestinal barrier, and regulating gut bacterial flora and serum metabolism. In conclusion, this study suggests that M-18C has the potential to be a new drug for the treatment of AKI.

## 1. Introduction

Acute kidney injury (AKI) is a disease consisting of the sudden loss of renal function and has become a common public health problem worldwide [1,2]. Common causes of AKI include ischemia, sepsis, and nephrotoxins. A characteristic pathological feature of AKI is renal tubular injury and cell death. The pathogenesis of AKI is very complex, and a specific mechanism has not been identified [3]. A close association between the kidneys and the gut has been reported in patients with AKI [4,5,6]. A range of inflammatory cytokines, such as tumor necrosis factor α (TNF-α) and interleukin 1β (IL-1β), are released in the bloodstream of patients with AKI, and these inflammatory factors are able to reduce the expression of Zonula occludens 1 (ZO-1) and Occludin in the gut, which can disrupt the intestinal barrier [5,7]. Impaired intestinal barrier integrity will increase the potential for intestinal contents to enter the bloodstream through the intestinal epithelium, exacerbating intestinal dysbiosis and leading to the entry of dysbiotic microbial metabolites into the bloodstream, exacerbating kidney injury [8]. The activation of TLR4 by LPS secreted by Gram-negative bacteria directly triggers the activation of NLRP3 inflammasome. The NLRP3 inflammasome, which consists of NLRP3, ASC, and pre-caspase-1, serves as an essential mediator of the inflammatory response in various AKI models [3]. Activation of the NLRP3 inflammasome has been shown to regulate the maturation and efflux of inflammatory cytokines, particularly interleukin 1β (IL-1β) and IL-18, which leads to an inflammatory response. Several previous studies have reported that the knockdown of NLRP3 exhibited a renoprotective effect in AKI mice [9]. Therefore, NLRP3 inflammatory vesicles are important therapeutic targets for preventing inflammatory responses associated with AKI.

In the present experiment, it has been demonstrated that M-18C is able to better inhibit the activity of monoacylglycerol lipase (MAGL), which is a serine hydrolase that catalyzes the conversion of long-chain monoacylglycerol to glycerol and fatty acids [6]. MAGL inhibitors have gained recognition as valuable agents in the treatment of various conditions including analgesia, anti-anxiety, and even anti-cancer. MAGL plays a key role in catalyzing the hydrolysis of endocannabinoid 2-alaxylon acylglycerol (2-AG) to generate pro-inflammatory substances [10,11]. In kidney disease, cannabinoid receptors are activated, expressed in immune cells, and have anti-inflammatory effects. Arachidonic amide and 2-AG are the two most studied endocannabinoids [12,13]. MAGL is widely found in many tissues, including the kidneys. MAGL inhibitors have been shown to alleviate ischemia–reperfusion injury by inhibiting the breakdown of 2-AG [14]. In addition, the inhibition of MAGL activity has been shown to alleviate neuroinflammation. Therefore, M-18C has a better potential for anti-inflammatory and AKI protection.

Our results provide evidence that M-18C has a kidney-protective effect and explore the intervention of M-18C in LPS-induced AKI by inhibiting NLRP3 inflammatory vesicles, repairing the intestinal barrier, and modulating the intestinal flora and serum metabolism. The results may provide a new therapeutic strategy for AKI.

## 2. Results

### 2.1. The Compound M-18C Functions as an Inhibitor of the Enzyme MAGL

**Figure 1:**
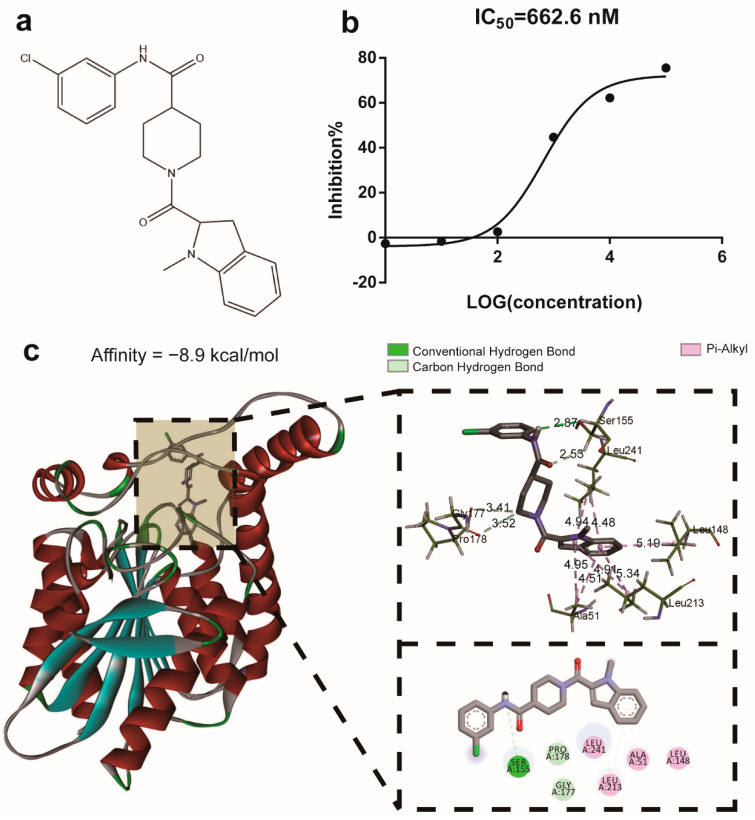
Molecular structure, IC50 values, and molecular docking of M-18C. The structure of M-18C (**a**), the inhibition kinetics of MAGL by M-18C (**b**), and the molecular docking findings for M-18C and MAGL (**c**).

The structure of the M-18C is depicted in Figure 1a. The molecular docking results showed that the binding energy of compound M-18C with the MAGL protein was −8.9 kcal/mol, suggesting that M-18C has a good binding ability with MAGL (Figure 1c). The IC50 value of compound M-18C for MAGL enzyme inhibition was determined to be 662.6 nM with a maximum inhibition rate of about 76% (Figure 1b). These results provide compelling evidence that M-18C is a potent inhibitor of MAGL.

### 2.2. M-18C Reduces the Levels of TNF-α and IL-1β in the Serum of Mice Caused by LPS

**Figure 2:**
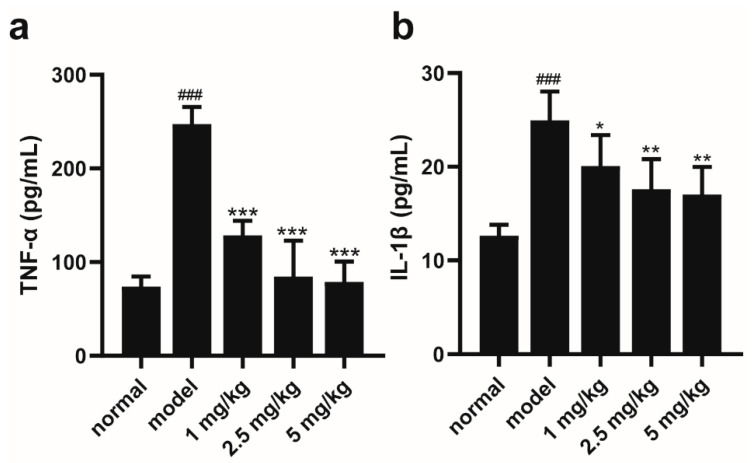
M-18C effects on the reduction in serum TNF-α and IL-1β levels in LPS-induced AKI mice. (**a**) TNF-α and (**b**) IL-1β levels in serum. Data were expressed as means ± SD (*n* = 6), ^###^ *p* < 0.001, compared with normal group; * *p* < 0.05, ** *p* < 0.01, *** *p* < 0.001 compared with model group.

ELISA results showed the serum level of TNF-α increased from 73.923 to 247.398 pg/mL in the model group compared with the normal group, and the serum TNF-α level decreased to 128.62, 84.63 and 78.99 pg/mL after the intervention of different concentrations of M-18C (1, 2.5 and 5 mg/kg), respectively (Figure 2a). Compared with the normal group, the serum level of IL-1β in the model group increased from 12.64 to 24.97 pg/mL. IL-1β expression in the serum of mice with different concentrations of M-18C (1, 2.5 and 5 mg/kg) decreased to 20.099, 17.604 and 17.038 pg/mL, respectively, compared with that in the model group (Figure 2b). This result demonstrates the ability of M-18C to reduce the expression levels of inflammatory factors in the serum of LPS-induced AKI mice.

### 2.3. M-18C Alleviates LPS-Induced AKI in Mice by Inhibiting NLRP3-Mediated Inflammation

**Figure 3:**
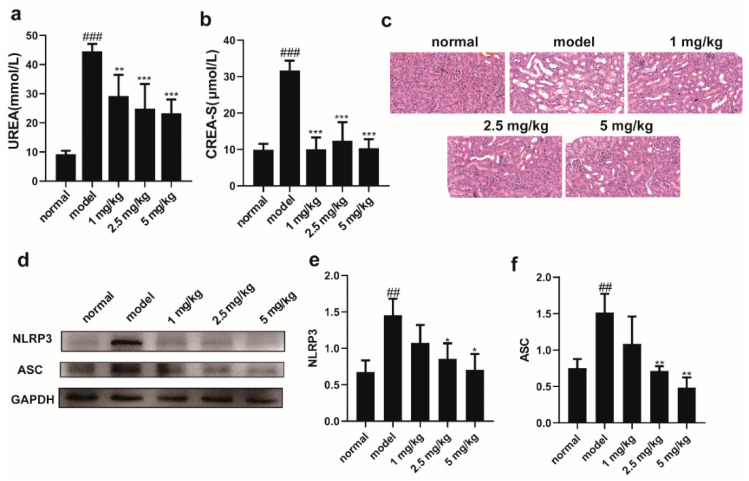
M-18C treatment protects the kidney in LPS-induced AKI mice. (**a**,**b**) Levels of UREA and CREA-S in serum. (**c**) Representative pictures of each group of kidney pathology (100×). (**d**) Expression levels of NLRP3 and ASC in the kidney were detected by Western blotting. The relative expression of ASC (**e**) and NLRP3 (**f**) was analyzed by image J. Data were expressed as means ± SD (*n* = 3 and 6), ^##^ *p* < 0.01, ^###^ *p* < 0.001, compared with normal group; * *p* < 0.05, ** *p* < 0.01, *** *p* < 0.001 compared with model group.

Elevated levels of UREA and CREA-S are two signs of impaired renal function. As illustrated in Figure 3a,b, the serum levels of UREA and CREA-S were significantly elevated in the model group compared to the normal group. This result suggests that renal injury occurred in the model group of mice. The expression levels of UREA and CREA-S in the serum of mice after M-18C intervention were significantly decreased compared with the model group (Figure 3a,b). The HE results showed that obvious edema and vacuoles appeared in the renal tubules of the mice in the model group, and the intervention of different concentrations of M-18C alleviated the pathological damage of the renal tissues of the mice to different degrees (Figure 3c). As shown in Figure 3d–f, it was found that the expression of NLRP3 and ASC proteins was hugely higher in the model group compared to the normal group, and both decreased to different degrees after drug administration. The above results suggest that M-18C reduces renal injury in LPS-induced AKI in mice by inhibiting the expression of NLRP3.

### 2.4. The Administration of M-18C Significantly Reduced NO, TNF-α, and IL-1β Levels and Downregulated NLRP3 and ASC Expression in LPS-induced RAW264.7

**Figure 4:**
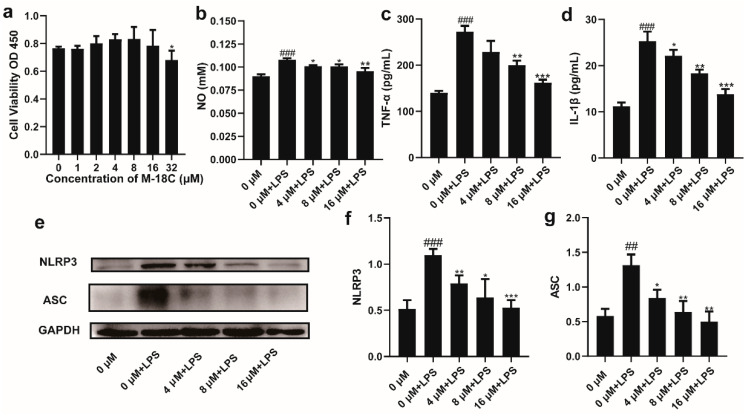
Effect of M-18C on the expression levels of NO, TNF-α and IL-1β in LPS-induced RAW264.7 cells. (**a**) Cells in different concentrations of M-18 c treatment after 24 h viability. Contents of (**b**) NO, (**c**) TNF-α and (**d**) IL-1β in RAW264.7 supernatant. (**e**) Expression levels of NLRP3, ASC and GAPDH as shown by Western blot. Analysis of (**f**) NLRP3 and (**g**) ASC expression levels by Image J. Data were expressed as means ± SD (*n* = 3 and 4), ^##^ *p* < 0.01, ^###^ *p* < 0.001, compared with 0 μM group; * *p* < 0.05, ** *p* < 0.01, *** *p* < 0.001 compared with LPS group.

The CCK-8 results showed that concentrations of M-18C up to 16 μM did not inhibit cell viability, but when the concentration increased to 32 μM, there was a significant decrease in cell viability (Figure 4a). The ELISA results showed that compared to the normal group, LPS significantly increased the levels of NO, TNF-α, and IL-1β in the supernatant of RAW264.7 cells. Compared to cells treated with LPS alone, the addition of M-18C significantly reduced the content of NO in the supernatant, with 16 μM showing the most significant effect. The levels of TNF-α and IL-1β in the supernatant also exhibited a gradient decrease after treatment with M-18C (Figure 4b–d). Western blot results showed that the levels of NLRP3 and ASC were significantly increased after LPS treatment, and they decreased with the dose of M-18C (Figure 4e–g). This result again suggests that M-18C is able to reduce the inflammation of LPS by inhibiting the NLRP3 signaling pathway.

### 2.5. M-18C Protects the Intestinal Barrier Caused by LPS

**Figure 5:**
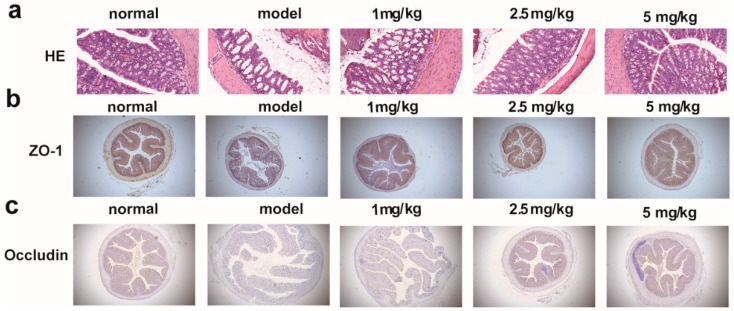
Effect of M-18C on colon of LPS-induced AKI in mice. Effect of M-18C on (**a**) Representative pictures of each group of colon pathology (100×). (**b**) ZO-1 and (**c**) Occludin protein expression in colonic tissues of mice with LPS-induced AKI detected by immunohistochemistry (10×).

In colonic tissue, intestinal permeability responds to the structural integrity of the intestinal barrier to some extent. HE results of colonic tissue showed altered morphology, intestinal mucosal edema and blurred borders of intestinal epithelial cells of mice in the model group, which gradually returned to normal after drug administration (Figure 5a). Immunohistochemical results likewise showed that the expression of ZO-1 and Occludin was significantly reduced in the model group and reversed to different degrees after drug administration (Figure 5b,c). The above results confirmed that M-18C could improve the intestinal barrier caused by LPS.

### 2.6. M-18C Regulates Imbalance of Gut Microbiota Caused by LPS

**Figure 6:**
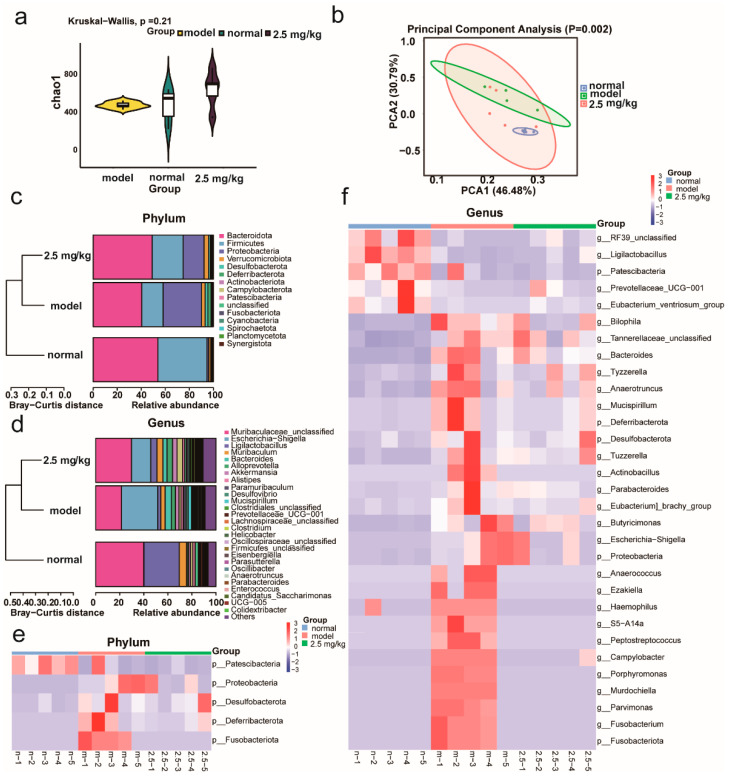
Effect of M-18C on intestinal flora of mice with LPS-induced AKI. (**a**) Analysis of alpha diversity by Chao1 index. (**b**) Results of principal component analysis (PCA) based on weighted UniFrac distance of 16S rRNA amplicon sequencing. (**c**,**d**) are the composition of the flora in the phylum and genus, respectively. Heat map of the differential flora among phylum (**e**) and genus (**f**).

We investigated the effect of M-18C on the intestinal flora of mice caused by LPS. From Figure 6, it could be seen that compared with the normal group, the diversity of intestinal flora of mice in the model group changed less, but the abundance changed more significantly, and there was a certain decline after drug administration. It was evident based on the beta analysis that there were changes in the species makeup of the gut microbiota in mice from the experimental group compared to those in the control group. Furthermore, following drug administration, a certain level of resemblance was observed between both groups. At the phylum level, the abundance of *Deferribacterota*, *Fusobacteriota*, *Proteobacteria*, *Desulfobacterota*, and *Patescibacteria* was notably higher in the model group compared to the normal group and decreased slightly after the administration of the drug. At the genus level, the abundance of the model groups *Eubacterium_brachy_group*, *Parabacteroides*, *Porphyromonas*, *Parvimonas*, *Bacteroides*, *Bilophila*, *Tuzzerella*, *Escherichia-Shigella*, *Mucispirillum*, *Fusobacterium*, *Tyzzerella*, *Butyricimonas*, and *Anaerotruncus* was significantly higher and *Ligilactobacillus* abundance was lower compared to the normal group, and all of them were ameliorated after administration. The above results showed that M-18C could balance the dysfunction of intestinal flora caused by LPS.

### 2.7. M-18C Regulates Serum Metabolism Caused by LPS

**Figure 7:**
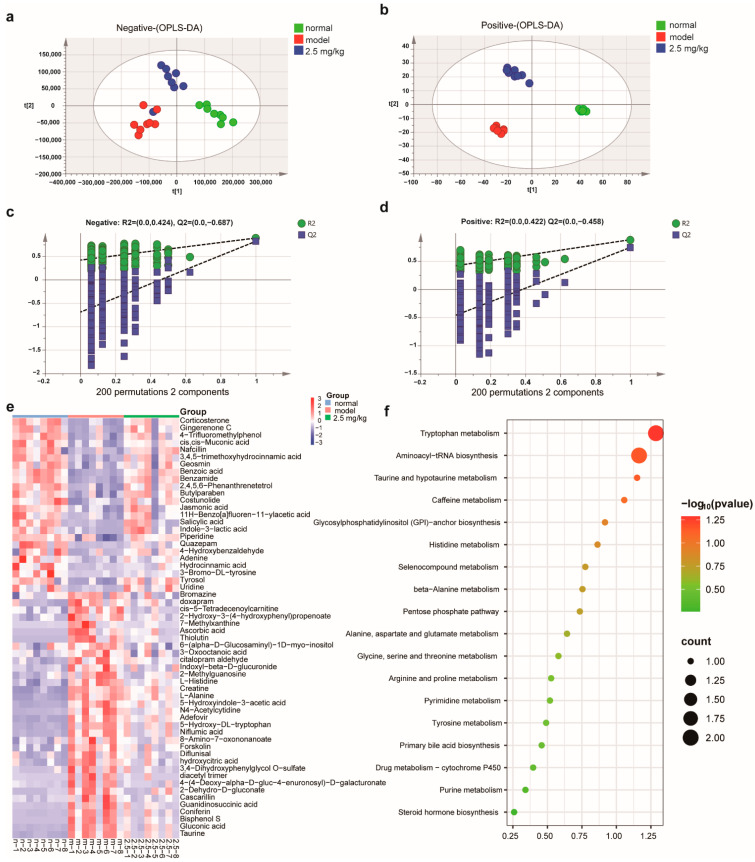
Effect of M-18C on serum metabolism in mice with LPS-induced AKI. (**a**) OPLS-DA examination was conducted on the negative ion profiles of serum samples from mice that received M-18C treatment following LPS-induced AKI. (**b**) OPLS-DA analysis to investigate positive ion profiles of serum samples from mice treated with M-18C. (**c**,**d**) Results derived from orthogonal experiments carried out in both positive and negative polarities. (**e**) Analysis of variations in metabolic activity. (**f**) Metabolic differential KEGG analysis.

In order to assess the effects of M-18C on serum metabolism in mice induced by LPS, we opted for the moderate dosage group (2.5 mg/kg) of M-18C and utilized LC-MS analysis to detect its metabolites that are present in the bloodstream. The application of OPLS-DA analysis revealed a distinct pattern of segregation among the control, model, and 2.5 mg/kg groups as depicted in Figure 7a,b. This suggests that M-18C has a significant impact on serum metabolism in mice with LPS-induced sepsis. The findings from orthogonal experiments indicated that the metabolic data exhibited consistent and dependable characteristics (Figure 7c,d). Subsequently, we conducted a comprehensive analysis of the compounds in each group (VIP > 1 and *p* < 0.05), as illustrated in Figure 7e. Our findings revealed a notable decrease in the levels of 25 compounds in the model group compared to those observed in the normal group, whereas 37 compounds exhibited significantly elevated levels. It is worth noting that these 62 compounds exhibited significant changes after administration, and their specific details can be found in Table 1. Next, the MetaboAnalyst database was utilized to conduct metabolic pathway enrichment analysis on 62 distinct metabolites. The outcomes of this analysis are depicted in Figure 7f. The results showed that M-18C could affect various amino acid metabolism, aminoacyl-tRNA biosynthesis, and metabolic pathways such as taurine metabolism in the serum caused by LPS.

## 3. Discussion

AKI is a common complication of some critical illnesses; however, there is no better therapeutic drug. Therefore, this experiment aims to investigate the therapeutic effect of M-18C on AKI and its specific mechanism. In our study, we observed the up-regulation of pro-inflammatory cytokines TNF-α and IL-1β in the serum of AKI mice, which was attenuated after M-18C treatment. Macrophages are phagocytic innate immune cells that play a key role in host defense and tissue homeostasis and are major contributors to the inflammatory response to AKI. The results of the in vitro experiments also demonstrated that M-18C was able to reduce the levels of TNF-α and IL-1β in the supernatants of LPS-induced RAW264.7 cells, suggesting that M-18C possesses a better anti-inflammatory activity. Blood biochemical indices showed that the intraperitoneal injection of LPS significantly increased the levels of UREA and CREA-S in the serum of mice, indicating impaired renal function. However, serum levels of UREA and CREA-S decreased in mice with AKI after treatment with M-18C. HE staining of the kidneys also showed that the renal tubules were over-dilated after LPS injection, and this dilatation was attenuated by M-18C injection, and these results indicated the protective effect of M-18C against LPS-induced AKI.

NLRP3 is an essential component of the inflammatory vesicle signaling pathway and can cooperate with ASC to ultimately trigger cellular pyrolysis [15,16]. Juan et al. demonstrated that the inhibition of NLRP3 expression in renal tissues attenuates renal injury in patients with AKI [15,16]. Wang et al. found that the MAGL inhibitor reduced inflammation by inhibiting the expression of NLRP3 in the rat brain [17]. In our experiments, we found that the expression of NLRP3 and ASC in the renal tissues of the model group of mice was significantly up-regulated, and this up-regulation was attenuated in a dose-dependent manner upon the administration of M-18C, while in vitro experiments again demonstrated that M-18C could reduce the expression of NLRP3 and ASC. It indicated that M-18C was able to attenuate LPS-induced AKI by inhibiting the expression of NLRP3 and ASC.

Research now suggests that the gut is associated with the development of numerous diseases and that disruption of the gut barrier may exacerbate systemic inflammation, leading to the worsening of AKI [8,18]. It has been demonstrated that LPS could cause damage to the intestinal architecture and disrupt the integrity of the intestinal barrier [19]. Pathological results showed a clear reduction in intestinal damage was observed in our experiments after M-18C treatment. The down-regulation of ZO-1 and Occludin, two commonly observed tight junction proteins in the intestinal tract, is indicative of the compromised integrity of the intestinal barrier [20,21]. And we found that the expression of ZO-1 and Occludin showed a significant rebound after M-18C treatment, indicating that M-18C protected the stability of the intestinal barrier in AKI mice. These findings suggest that M-18C mitigates intestinal injury and fortifies the integrity of the intestinal barrier.

Studies have shown that dysbiosis of the gut microbiota could exacerbate AKI [22,23]. Therefore, we utilized the 16S rRNA technique to examine the gut flora of each group of mice. At the phylum level, five species of gut bacteria were significantly elevated in abundance in the model group, and the M-18C intervention was shown to reduce their abundance. At the genus level, significant changes in gut bacteria also occurred in the model group, which were significantly dialed back by the M-18C intervention. This demonstrated that M-18C was able to have a better regulatory effect on the disorders of intestinal bacteria in model mice. *Desulfobacterota* and *Proteobacteria* have been reported to be able to accelerate the progression of inflammatory diseases by activating inflammatory vesicle signaling pathways [24], and M-18C was able to reduce the abundance of *Desulfobacterota* and *Proteobacteria* in the intestinal tract of AKI mice. There is evidence that *Deferribacterota* and *Mucispirillum* are strongly associated with intestinal inflammation and the risk of kidney disease [25,26,27,28], whereas in our experiments, we found that *Deferribacterota* abundance was elevated in the intestinal tracts of AKI mice, and M-18C intervention significantly reduced *Deferribacterota* abundance. Wei et al. demonstrated that the abundance of *Tuzzerella* was positively correlated with urea nitrogen, reactive oxygen species, and IL-1β in the serum of hyperuricemic mice [29,30], and M-18C was able to reduce the abundance of *Tuzzerella* in the intestines of AKI mice. In conclusion, M-18C has a good regulatory effect on the disturbance of intestinal flora in AKI mice caused by LPS.

The LC-MS results revealed that M-18C exhibited a significant correction on 62 metabolites, indicating its regulatory effect on metabolic disorders in mice with LPS-induced AKI. The KEGG analysis indicated a significant enrichment of the pathways involved in ammonyl-tRNA biosynthesis and the metabolism of alanine, aspartate, and glutamate. These pathways have been previously shown to exhibit anti-inflammatory effects and regulate the expression of proteins associated with NLRP3 [31,32]. The remaining samples also exhibited a significant increase in indole-3-lactic acid levels following administration, which was observed to effectively ameliorate colonic inflammation and down-regulate NLRP3 expression [33]. The level of Costunolide was observed to decrease with LPS treatment, but it increased significantly after administration. In addition, Costunolide has the ability to target NLRP3 therapeutically, effectively alleviating related inflammatory diseases [34]. The above results demonstrate that M-18C could downregulate NLRP3 expression by modulating metabolism.

## 4. Materials and Methods

### 4.1. Drugs and Reagents

MAGLZ-18(c) (M-18C) (purity > 98%) was purchased from Shandong New Time Pharmaceutical Co. LTD. (Linyi, China). LPS was purchased from Sigma-Aldrich (Shanghai, China). Antibodies targeting ZO-1, Occludin, and GAPDH were obtained from Abcam Technology (Cambridge, UK). The antibodies against NLRP3 and ASC were procured from Cell Signaling Technology (Boston, MA, USA). HRP-labeled goat anti-mouse, HRP-labeled goat anti-rabbit, chemiluminescent HRP substrate and BeyoECL Plus were obtained by Beyotime technology (Shanghai, China). Polyvinylidene fluoride (PVDF) membranes were obtained from Merck Millipore (MD, USA). A Hematoxylin–Eosin (H&E) Staining Kit was purchased from Beijing Solarbio Science & Technology Co. Ltd. (Beijing, China).

### 4.2. Establishment of a Mouse Model of LPS-Induced AKI

A total of 50 C57BL/6J male mice (6–8 weeks old, 18–22 g) were purchased from the Beijing Vital River Laboratory Animal Technology Co., Ltd. (Beijing, China) (License Number: SCXK (Beijing) 2019-0006). All mice were kept in a controlled environment with a consistent room temperature of 22 ± 2 °C and relative humidity maintained at 60 ± 5%. The light–dark cycle adhered to a 12 h pattern, while the mice had unlimited access to tap water and pelleted feed (Certificate No. AN-IACUC-2023-044).

After 1 week of acclimatization, the mice were randomly divided into five groups (including normal, model, M-18C 1, 2.5 and 5 mg/kg groups). In the experiment, saline was injected intraperitoneally according to weight in the normal group, while the model group and the drug administration group were administered LPS according to weight. One hour later, saline was injected into the normal group, and 25 mg/kg LPS was injected into the model and drug-administered groups according to body weight. Mice were dissected after 24 h, blood was taken from the main abdominal vein, and the kidney and colon were taken and frozen at −80 °C. Stools were obtained from the colon and stored in liquid nitrogen for intestinal flora analysis.

### 4.3. IC50 Determination of MAGL Inhibitor

The Monoacylglycerol Lipase Inhibitor Screening Assay Kit (Cayman, Ann Arbor, MI, USA) was employed for the experiment. Following the instructions provided in the kit manual, Assay Buffer was added to each well. Subsequently, an appropriate amount of MAGL, solvent and samples were gently introduced into each well as per the instructions before, allowing MAGL to bind with the inhibitor for a duration of 15 min. After the initiation of the MAGL substrate reaction in each well, absorbance at 410 nm was measured after a duration of 10 min. The obtained results were plotted using GraphPad Prism 8.0 software, and IC50 values were calculated.

### 4.4. Molecular Docking

The UniProt database (https://www.uniprot.org/) accessed on 15 February 2023 was utilized for searching and retrieving the PDB format of the large molecule MAGL protein 5ZUN, which was subsequently imported into AutoDock Tools 1.5.6 to eliminate solvent and excess ligands, which was followed by hydrogenation calculations and conversion to the PDBQT format. Subsequently, a grid box was established using small molecules also converted to the PDBQT format after adjusting parameters for rigid docking. The resulting docking outcomes were visualized in Discovery Studio 4.5 Client to identify binding sites between the large and small molecules with subsequent image export.

### 4.5. Cell Lines and Cell Culture

The RAW264.7 cell line (a mouse monocyte macrophage leukemia cell line) was acquired from the Chinese Academy of Sciences in Shanghai, China. The cells were cultured in Dulbecco’s Modified Eagle Medium (DMEM) supplemented with 10% fetal bovine serum (FBS) and 1% penicillin–streptomycin solution (100 units/mL). Maintenance of the cells was carried out in a CO_2_ incubator at a temperature of 37 °C.

### 4.6. Cell Viability Assessment

The RAW264.7 cells were cultured in a 96-well plate with a seeding density of 10,000 cells per well and incubated overnight in a cell culture incubator. Following a 24 h exposure to M-18C, each well was supplemented with 10 μL of CCK-8 solution (Beyotime technology, Shanghai, China) and incubated for approximately 30–60 min. Subsequently, the optical density (OD) values were assessed at 450 nm using a microplate reader manufactured by Bio Tek located in Winooski, VT, USA to determine the percentage of viable cells.

### 4.7. Determination of NO

The RAW264.7 cells were cultured in a 96-well plate at a seeding density of 10,000 cells per well and incubated overnight in a cell culture incubator. The control group was treated with an equal volume of medium, while the remaining groups were treated with LPS to a concentration of 1 μg/mL for 1 h before being treated with different concentrations of M-18C. After 24 h, the supernatant was collected, and the NO content was measured according to the requirements outlined in the Griess Reagent System Technical Bulletin (Promega, Beijing, China).

### 4.8. Western Blotting Assay

Lysates from sterile kidneys and RAW264.7 were separated using the 12% SDS-PAGE (sodium dodecyl sulfate-polyacrylamide gel electrophoresis) technique and subsequently transferred onto membranes made of polyvinylidene difluoride. The membranes were sealed tightly by utilizing skim milk powder (5%) for 1 h at ambient temperature and subsequently incubated overnight with the primary antibody at a refrigerated temperature of 4 °C. Afterwards, the membranes underwent a 2 h incubation at a temperature of 37 °C with the HRP secondary antibody. The blot bands were subsequently detected using reagents for enhanced chemiluminescence and captured by an imager (Chemi Scope 6200, Clinx Scientific Instruments Co., Ltd., Shanghai, China). Ultimately, grayscale values were calculated using Image J software to facilitate quantitative evaluation.

### 4.9. Enzyme-Linked Immunosorbent Assay (ELISA)

The levels of TNF-α and IL-1β in serum were determined by enzyme-linked immunosorbent assay (ELISA) kits (Beyotime, Shanghai, China) according to the manufacturer’s instructions.

### 4.10. Blood Biochemistry

The serum was collected and assayed for urea (UREA) and creatinine (CREA-S) according to the instructions of the kit (Mindrary, Shanghai, China).

### 4.11. Hematoxylin and Eosin (HE) Staining

Thin sections of kidney and colon tissues that had been embedded in paraffin were dehydrated using a series of graded ethanol before being stained with H&E. Subsequently, three pathologists evaluated the extent of tissue inflammation and damage.

### 4.12. Immunohistochemistry (IHC)

After conducting gradient dewaxing using a combination of xylene and ethanol, the kidney and colon tissue sections were subjected to high-pressure heating in a solution containing sodium citrate. Following this, the sections underwent a ten-minute treatment with 3% hydrogen peroxide, which was followed by three rinses using PBS. Additionally, the sections were exposed to 5% normal goat serum for ten minutes and then rinsed three more times with PBS. Next, the sections were subjected to overnight incubation at 4 °C with a primary antibody (1:250). After undergoing three PBS rinses, the sections were subjected to treatment with a secondary antibody conjugated with biotin and streptavidin–horseradish peroxidase at room temperature. Subsequently, they underwent three additional rinses with PBS before being subjected to diaminobenzidine (DAB) staining provided by Beijing Daylight Technology Co., Ltd. Following the staining procedure, the sections were dehydrated and coated with a neutral resin. The target proteins were observed in an arbitrary manner using a microscope. The intensity of staining and proportion of cells exhibiting positive results were assessed and analyzed utilizing Image J software (version 1.5.7, National Institutes of Health).

### 4.13. Examining the Influence of M-18C on Serum Metabolism Caused by LPS via Unbiased LC-MS Analysis

Prepare 100 μL serum samples per mouse and transfer them to Eppendorf tubes with internal standards. Use a mixture of methanol and water (4:1 *v*/*v*) that has been chilled on ice. Store the serum samples at −20 °C for 30 min, then centrifuge them at 13,000 rpm for 15 min at a temperature of 4 °C. Next, add a methanol and water mixture (1:4, *v*/*v*) to each sample followed by vortexing for 30 s. Allow the samples to rest at −20 °C for two hours before analyzing their metabolic profiles using a Dionex Ultimate 3000 RS ultra-performance liquid chromatography system equipped with a Q Exactive quadrupole orbital mass spectrometer and heated electrospray ionization (ESI) source from Thermo Fisher Scientific in Waltham, MA, USA.

### 4.14. 16S rRNA Gene Sequencing

The DNA samples from different fecal groups were extracted using the E.Z.N.A.^®^ Stool DNA Kit (Omega Biotek, Norcross, GA, USA) in accordance with the manufacturer’s provided guidelines. Quality assessment of DNA extraction was conducted through agarose gel electrophoresis, while quantification was performed using a UV spectrophotometer. The DNA obtained was diluted to a concentration of 1 ng/μL and used as the initial material for PCR amplification targeting the bacterial 16S rRNA gene. In this process, barcode primers and Takara Ex Taq (Takara) were employed. The universal primers, specifically 343 F and 798 R, were utilized to amplify the V3–V4 variable region of the 16S rRNA gene. Amplicon quality was assessed using gel electrophoresis, followed by purification with AMPure XP beads (Agencourt), and an additional round of PCR amplification. After further purification with AMPure XP beads, quantification was performed using the Qubit dsDNA Detection Kit. Finally, equal amounts of purified amplicons were pooled for subsequent sequencing analysis.

### 4.15. Statistical Analysis

All data were expressed as the average ± SD. The differences between the various groups were assessed using one-way analysis of variance (ANOVA), which was followed by a post hoc Bonferroni correction for multiple comparisons. Statistical significance was defined as *p* < 0.05.

## 5. Conclusions

In conclusion, M-18C was able to reduce the release of TNF-α and IL-1β by inhibiting the expression of NLRP3 and ASC proteins, repairing the intestinal barrier, and regulating the intestinal flora and serum metabolism, thereby alleviating LPS-induced AKI. This suggests that M-18C is a promising potential drug for the treatment of AKI. This finding also indicates the potential of MAGL inhibitors in safeguarding against LPS-induced AKI, thereby offering a novel avenue for further exploration in MAGL inhibitor research.

## Figures and Tables

**Table 1:** Details of metabolic differentials among three groups.

No.	Name	Formula	RT (min)	Ion Mod	VIP	Model vs. Normal	2.5 mg/kg vs. Model
1	11H-Benzo[a]fluoren-11-ylacetic acid	C_19_H_14_O_2_	4.714	ESI−	1.23099	↓^##^	↑*
2	2,4,5,6-Phenanthrenetetrol	C_14_H_10_O_4_	8.463	ESI−	1.50203	↓^####^	↑*
3	2-Dehydro-D-gluconate	C_6_H_10_O_7_	1.202	ESI−	1.33805	↑^###^	↓*
4	2-Hydroxy-3-(4-hydroxyphenyl-propenoate	C_9_H_8_O_4_	1.882	ESI−	1.59186	↑^##^	↓**
5	2-Methylguanosine	C_11_H_15_N_5_O_5_	4.949	ESI−	1.57437	↑^##^	↓**
6	3,4,5-trimethoxyhydrocinnamic acid	C_12_H_16_O_5_	10.617	ESI−	1.45642	↓^####^	↑*
7	3,4-Dihydroxyphenylglycol O-sulfate	C_8_H_10_O_7_S	1.612	ESI−	1.42525	↑^##^	↓*
8	3-Bromo-DL-tyrosine	C_9_H_10_BrNO_3_	5.655	ESI−	1.02757	↓^#^	↑*
9	3-Oxooctanoic acid	C_8_H_14_O_3_	5.061	ESI−	1.02523	↑^##^	↓*
10	4-(4-Deoxy-alpha-D-gluc-4-enuronosyl)-D-galacturonate	C_12_H_16_O_12_	1.167	ESI−	1.40788	↑^##^	↓*
11	4-Hydroxybenzaldehyde	C_7_H_6_O_2_	4.433	ESI−	1.01709	↓^#^	↑*
12	4-Trifluoromethylphenol	C_7_H_5_F_3_O	3.941	ESI−	1.45253	↓^####^	↑*
13	5-Hydroxy-DL-tryptophan	C_11_H_12_N_2_O_3_	3.802	ESI−	1.46735	↑^####^	↓*
14	5-Hydroxyindole-3-acetic acid	C_10_H_9_NO_3_	4.407	ESI−	1.47610	↑^####^	↓*
15	6-(alpha-D-Glucosaminyl)-1D-myo-inositol	C_12_H_23_NO_10_	1.378	ESI−	1.47230	↑^#^	↓**
16	7-Methylxanthine	C_6_H_6_N_4_O_2_	1.854	ESI−	1.31582	↑^#^	↓*
17	8-Amino-7-oxononanoate	C_9_H_17_NO_3_	6.475	ESI−	1.17585	↑^##^	↓*
18	Adefovir	C_8_H_12_N_5_O_4_P	6.24	ESI−	1.52359	↑^####^	↓*
19	Adenine	C_5_H_5_N_5_	6.998	ESI−	1.05053	↓^#^	↑*
20	Bisphenol S	C_12_H_10_O_4_S	1.316	ESI−	1.44627	↑^##^	↓*
21	Bromazine	C_17_H_20_BrNO	5.243	ESI−	1.31946	↑^#^	↓*
22	Butylparaben	C_11_H_14_O_3_	10.001	ESI−	1.62456	↓^##^	↑**
23	Cascarillin	C_22_H_32_O_7_	8.64	ESI−	1.27469	↑^#^	↓*
24	cis,cis-Muconic acid	C_6_H_6_O_4_	6.379	ESI−	1.35960	↓^##^	↑*
25	cis-5-Tetradecenoylcarnitine	C_21_H_39_NO_4_	12.571	ESI−	1.67272	↑^###^	↓***
26	Citalopram aldehyde	C_18_H_14_FNO_2_	7.431	ESI−	1.13161	↑^##^	↓*
27	Coniferin	C_16_H_22_O_8_	7.579	ESI−	1.26308	↑^###^	↓*
28	Corticosterone	C_21_H_30_O_4_	11.773	ESI−	1.50496	↓^####^	↑**
29	Costunolide	C_15_H_20_O_2_	11.187	ESI−	1.31224	↓^##^	↑*
30	Creatine	C_4_H_9_N_3_O_2_	1.427	ESI−	1.70032	↑^####^	↓****
31	Diacetyl trimer	C_12_H_18_O_6_	1.653	ESI−	1.28257	↑^##^	↓*
32	Diflunisal	C_13_H_8_F_2_O_3_	1.809	ESI−	1.27618	↑^##^	↓*
33	Doxapram	C_24_H_30_N_2_O_2_	7.239	ESI−	1.31590	↑^##^	↓*
34	Forskolin	C_22_H_34_O_7_	8.29	ESI−	1.34054	↑^###^	↓*
35	Geosmin	C_12_H_22_O	11.826	ESI−	1.27614	↓^##^	↑*
36	Gingerenone C	C_20_H_22_O_4_	10.646	ESI−	1.35761	↓^###^	↑*
37	Gluconic acid	C_6_H_12_O_7_	1.219	ESI−	1.41214	↑^###^	↓*
38	Guanidinosuccinic acid	C_5_H_9_N_3_O_4_	1.200	ESI−	1.31446	↑^##^	↓*
39	Hydrocinnamic acid	C_9_H_10_O_2_	7.034	ESI−	1.15891	↓^##^	↑*
40	Hydroxycitric acid	C_6_H_8_O_8_	1.169	ESI−	1.41456	↑^###^	↓**
41	Indole-3-lactic acid	C_11_H_11_NO_3_	4.963	ESI−	1.45166	↓^###^	↑**
42	Indoxyl-beta-D-glucuronide	C_14_H_15_NO_7_	3.785	ESI−	1.15719	↑^##^	↓*
43	Jasmonic acid	C_12_H_18_O_3_	10.665	ESI−	1.44216	↓^###^	↑*
44	L-Histidine	C_6_H_9_N_3_O_2_	1.448	ESI−	1.59545	↑^####^	↓**
45	N4-Acetylcytidine	C_11_H_15_N_3_O_6_	5.189	ESI−	1.42193	↑^###^	↓*
46	Nafcillin	C_21_H_22_N_2_O_5_S	10.423	ESI−	1.14129	↓^#^	↑*
47	Niflumic acid	C_13_H_9_F_3_N_2_O_2_	1.507	ESI−	1.49980	↑^####^	↓*
48	Quazepam	C_17_H_11_ClF_4_N_2_S	1.892	ESI−	1.26974	↓^###^	↑*
49	Salicylic acid	C_7_H_6_O_3_	5.038	ESI−	1.32580	↓^###^	↑*
50	Taurine	C_2_H_7_NO_3_S	1.318	ESI−	1.46605	↑^####^	↓*
51	Thiolutin	C_8_H_8_N_2_O_2_S_2_	1.502	ESI−	1.27014	↑^#^	↓*
52	Tyrosol	C_8_H_10_O_2_	8.738	ESI−	1.51769	↓^##^	↑**
53	Uridine	C_9_H_12_N_2_O_6_	3.061	ESI−	1.01969	↓^#^	↑**
54	Ascorbic acid	C_6_H_8_O_6_	0.910	ESI+	3.63786	↑^##^	↓*
55	Benzamide	C_7_H_7_NO	3.990	ESI+	1.23133	↓^####^	↑***
56	Benzoic acid	C_7_H_6_O_2_	4.250	ESI+	9.38332	↓^####^	↑***
57	Creatine	C_4_H_9_N_3_O_2_	0.916	ESI+	9.34523	↑^####^	↓*
58	Gluconic acid	C_6_H_12_O_7_	0.772	ESI+	2.19480	↑^###^	↓*
59	L-Alanine	C_3_H_7_NO_2_	0.917	ESI+	1.63292	↑^####^	↓*
60	Piperidine	C_5_H_11_N	10.42	ESI+	2.02758	↓^##^	↑*
61	Taurine	C_2_H_7_NO_3_S	0.812	ESI+	4.30360	↑^####^	↓*
62	Thiolutin	C_8_H_8_N_2_O_2_S_2_	0.948	ESI+	1.07162	↑^####^	↓**

Data were expressed as means ± SD (*n* = 6), ^#^ *p* < 0.05, ^##^ *p* < 0.01, ^###^ *p* < 0.001, ^####^ *p* < 0.0001, compared with normal group; * *p* < 0.05, ** *p* < 0.01, *** *p* < 0.001, **** *p* < 0.0001, compared with model group.

## Data Availability

The data that support the findings of this study are available from the corresponding author upon reasonable request.

## References

[1] Guo Y., Wu B., Chen Q., Min S. (2022). Parecoxib ameliorates renal toxicity and injury in sepsis-induced mouse model and LPS-induced HK-2 cells. Drug Dev. Res..

[2] Prescott H.C., Angus D.C. (2018). Enhancing Recovery from Sepsis: A Review. JAMA.

[3] Bullen A.L., Ix J.H. (2020). Is Tubular Dysfunction a Risk Factor for AKI?. Nephron.

[4] Duncan C.F., Youngstein T., Kirrane M.D., Lonsdale D.O. (2021). Diagnostic Challenges in Sepsis. Curr. Infect. Dis. Rep..

[5] Adib-Conquy M., Cavaillon J.M. (2007). Stress molecules in sepsis and systemic inflammatory response syndrome. FEBS Lett..

[6] Krivan S., Kapelouzou A., Vagios S., Tsilimigras D.I., Katsimpoulas M., Moris D., Aravanis C.V., Demesticha T.D., Schizas D., Mavroidis M. (2019). Increased expression of Toll-like receptors 2, 3, 4 and 7 mRNA in the kidney and intestine of a septic mouse model. Sci. Rep..

[7] Chao L.K., Lin C.H., Chiu H.W., Wong W.T., Chiu H.W., Tasi Y.L., Kuo Y.H., Chiu Y.C., Liu M.L., Ho C.L. (2015). Peroxyauraptenol Inhibits Inflammation and NLRP3 Inflammasome Activation by Inhibiting Reactive Oxygen Species Generation and Preserving Mitochondrial Integrity. J. Agric. Food Chem..

[8] Zhang J., Ankawi G., Sun J., Digvijay K., Yin Y., Rosner M.H., Ronco C. (2018). Gut-kidney crosstalk in septic acute kidney injury. Crit. Care.

[9] Kim S.M., Kim Y.G., Kim D.J., Park S.H., Jeong K.H., Lee Y.H., Lim S.J., Lee S.H., Moon J.Y. (2018). Inflammasome-Independent Role of NLRP3 Mediates Mitochondrial Regulation in Renal Injury. Front. Immunol..

[10] Dinh T.P., Freund T.F., Piomelli D. (2002). A role for monoglyceride lipase in 2-arachidonoylglycerol inactivation. Chem. Phys. Lipids.

[11] Pagano E., Borrelli F., Orlando P., Romano B., Monti M., Morbidelli L., Aviello G., Imperatore R., Capasso R., Piscitelli F. (2017). Pharmacological inhibition of MAGL attenuates experimental colon carcinogenesis. Pharmacol. Res..

[12] Karwad M.A., Couch D.G., Theophilidou E., Sarmad S., Barrett D.A., Larvin M., Wright K.L., Lund J.N., O’Sullivan S.E. (2017). The role of CB(1) in intestinal permeability and inflammation. FASEB J..

[13] Moradi H., Oveisi F., Khanifar E., Moreno-Sanz G., Vaziri N.D., Piomelli D. (2016). Increased Renal 2-Arachidonoylglycerol Level Is Associated with Improved Renal Function in a Mouse Model of Acute Kidney Injury. Cannabis Cannabinoid Res..

[14] Çakır M., Aydın A., Tekin S. (2023). Protective effect of fatty acid amide hydrolase inhibitor URB597 and monoacylglycerol lipase inhibitor KML29 on renal ischemia–reperfusion injury via toll-like receptor 4/nuclear factor-kappa B pathway. Int. Immunopharmacol..

[15] Chen R., Xu H., Guo Z., Zhang P., Chen J., Chen Z. (2022). CID16020046, a GPR55 antagonist, attenuates sepsis-induced acute kidney injury. Mol. Med. Rep..

[16] Privratsky J.R., Ide S., Chen Y., Kitai H., Ren J., Fradin H., Lu X., Souma T., Crowley S.D. (2023). A macrophage-endothelial immunoregulatory axis ameliorates septic acute kidney injury. Kidney Int..

[17] Wang C.Q., Su Z., Dai C.G., Song J.L., Qian B. (2023). Multi-omics analysis reveals BDE47 induces depression-like behaviors in mice by interfering with the 2-arachidonoyl glycerol-associated microbiota-gut-brain axis. Ecotoxicol. Environ. Saf..

[18] Saranya G.R., Viswanathan P. (2023). Gut microbiota dysbiosis in AKI to CKD transition. Biomed. Pharmacother..

[19] He Y., Xu M., Lu S., Zou W., Wang Y., Fakhar E.A.K.M., Iqbal M., Li K. (2023). Seaweed polysaccharides treatment alleviates injury of inflammatory responses and gut barrier in LPS-induced mice. Microb. Pathog..

[20] Krause G., Winkler L., Mueller S.L., Haseloff R.F., Piontek J., Blasig I.E. (2008). Structure and function of claudins. Biochim. Biophys. Acta.

[21] Duan Y., Huang J., Sun M., Jiang Y., Wang S., Wang L., Yu N., Peng D., Wang Y., Chen W. (2023). Poria cocos polysaccharide improves intestinal barrier function and maintains intestinal homeostasis in mice. Int. J. Biol. Macromol..

[22] Xu Y., Kong X., Zhu Y., Xu J., Mao H., Li J., Zhang J., Zhu X. (2022). Contribution of gut microbiota toward renal function in sepsis. Front. Microbiol..

[23] Kobayashi T., Iwata Y., Nakade Y., Wada T. (2021). Significance of the Gut Microbiota in Acute Kidney Injury. Toxins.

[24] Zmora N., Levy M., Pevsner-Fishcer M., Elinav E. (2017). Inflammasomes and intestinal inflammation. Mucosal Immunol..

[25] Yang Y., Yu J., Huo J., Yan Y. (2023). Sesamolin Attenuates Kidney Injury, Intestinal Barrier Dysfunction, and Gut Microbiota Imbalance in High-Fat and High-Fructose Diet-Fed Mice. J. Agric. Food Chem..

[26] Huang J., Jiang T., Kang J., Xu J., Dengzhang Y., Zhao Z., Yang C., Wu M., Xu X., Zhang G. (2022). Synergistic Effect of Huangqin Decoction Combined Treatment with Radix *Actinidiae chinensis* on DSS and AOM-Induced Colorectal Cancer. Front. Pharmacol..

[27] Herp S., Durai Raj A.C., Salvado Silva M., Woelfel S., Stecher B. (2021). The human symbiont *Mucispirillum schaedleri*: Causality in health and disease. Med. Microbiol. Immunol..

[28] Loy A., Pfann C., Steinberger M., Hanson B., Herp S., Brugiroux S., Gomes Neto J.C., Boekschoten M.V., Schwab C., Urich T. (2017). Lifestyle and Horizontal Gene Transfer-Mediated Evolution of *Mucispirillum schaedleri*, a Core Member of the Murine Gut Microbiota. mSystems.

[29] Wei B., Ren P., Xue C., Wang Y., Tang Q. (2023). Guluronate oligosaccharides exerts beneficial effects on hyperuricemia and regulation of gut microbiota in mice. Food Biosci..

[30] Gao J., Xu K., Liu H., Liu G., Bai M., Peng C., Li T., Yin Y. (2018). Impact of the Gut Microbiota on Intestinal Immunity Mediated by Tryptophan Metabolism. Front. Cell. Infect. Microbiol..

[31] Chao L., Lin J., Zhou J., Du H., Chen X., Liu M., Qu Q., Lv W., Guo S. (2022). Polyphenol Rich *Forsythia suspensa* Extract Alleviates DSS-Induced Ulcerative Colitis in Mice through the Nrf2-NLRP3 Pathway. Antioxidants.

[32] Wang J., Wang X., Ma X., Xu B., Chen L., Chen C., Liu W., Liu Y., Xiang Z. (2022). Therapeutic effect of *Patrinia villosa* on TNBS-induced ulcerative colitis via metabolism, vitamin D receptor and NF-kappaB signaling pathways. J. Ethnopharmacol..

[33] Cui Q., Zhang Z., Tian X., Liang X., Lu Y., Shi Y., Kuerman M., Wang R., Yu Z., Gong P. (2023). Bifidobacterium bifidum Ameliorates DSS-Induced Colitis in Mice by Regulating AHR/NRF2/NLRP3 Inflammasome Pathways through Indole-3-lactic Acid Production. J. Agric. Food Chem..

[34] Xu H., Chen J., Chen P., Li W., Shao J., Hong S., Wang Y., Chen L., Luo W., Liang G. (2023). Costunolide covalently targets NACHT domain of NLRP3 to inhibit inflammasome activation and alleviate NLRP3-driven inflammatory diseases. Acta Pharm. Sin. B.

